# Preservation and recovery of mangrove ecosystem carbon stocks in abandoned shrimp ponds

**DOI:** 10.1038/s41598-019-54893-6

**Published:** 2019-12-04

**Authors:** Angie Elwin, Jacob J. Bukoski, Vipak Jintana, Elizabeth J. Z. Robinson, Joanna M. Clark

**Affiliations:** 1 Department of Geography and Environmental Science, University of Reading, Reading, UK; 2 Department of Environmental Science, Policy and Management, University of California, Berkeley, CA, USA; 3 Faculty of Forestry, Kasetsart University, Bangkok, Thailand; 4 School of Agriculture, Policy and Development, University of Reading, Reading, UK

**Keywords:** Climate-change mitigation, Ecosystem services, Environmental impact

## Abstract

Mangrove forests capture and store exceptionally large amounts of carbon and are increasingly recognised as an important ecosystem for carbon sequestration. Yet land-use change in the tropics threatens this ecosystem and its critical ‘blue carbon’ (carbon stored in marine and coastal habitats) stores. The expansion of shrimp aquaculture is among the major causes of mangrove loss globally. Here, we assess the impact of mangrove to shrimp pond conversion on ecosystem carbon stocks, and carbon losses and gains over time after ponds are abandoned. Our assessment is based on an intensive field inventory of carbon stocks at a coastal setting in Thailand. We show that although up to 70% of ecosystem carbon is lost when mangroves are converted to shrimp ponds, some abandoned ponds contain deep mangrove soils (>2.5 m) and large carbon reservoirs exceeding 865 t carbon per hectare. We also found a positive recovery trajectory for carbon stocks in the upper soil layer (0–15 cm) of a chronosequence of abandoned ponds, associated with natural mangrove regeneration. Our data suggest that mangrove carbon pools can rebuild in abandoned ponds over time in areas exposed to tidal flushing.

## Introduction

Mangrove forests are highly productive ecosystems^1–3^ that are increasingly recognised as major hotspots for global carbon (C) sequestration and burial^2,4^. On average, mangroves have a mean whole-ecosystem C stock of ~950 t C ha^−1^ ^2^, which is around 2.5–5 times higher than the mean ecosystem C stock found in temperate, boreal and upland tropical forests (200–400 t C ha^−1^ ^5^).

Ecosystem C storage in mangroves worldwide is dominated by belowground soil C pools^6–8^, the variability of which is driven mainly by tidal amplitude and minimum temperature^9^. Organic-rich deposits can form up to several meters deep and can be stored below-ground for centuries if left undisturbed^10,11^. Globally, the C stored by mangroves (4.0–20 Pg C^6^) is equivalent to over twice the annual global anthropogenic CO_2_ emissions^12^, highlighting the potential role of mangrove conservation for climate change mitigation^13^.

Mangrove forests are also one of the world’s most threatened ecosystems^14,15^. Approximately 30–50% of global mangrove cover has been lost over the last 50 years due to urbanisation and the demand for alternative land uses^16,17^. Shrimp aquaculture is one such land-use change substantially driving global mangrove loss^17–19^. This problem has been particularly acute in Thailand where extensive areas of mangrove were replaced with aquaculture ponds during the 1980s–2000s^20^. Mangrove cover was reduced from 370,000 ha in 1961 to 167,500 ha in 1996, around half of this because of aquaculture^19^.

Conversion of mangroves into shrimp ponds has the potential to reverse the C sink function of the forests^21^ because pond construction changes the topography of the land, therefore altering key biophysical variables controlling CO_2_ flux from the soils, such as soil temperature, soil moisture content, and the duration of tidal inundation^22–24^. The construction of shrimp ponds results in the removal of trees and around 1.5 m of the top layer of soil, resulting in loss of significant amounts of C stored in the vegetation (trees aboveground and belowground roots), in the litter, and part of the soil. The excavated soil is usually piled up under aerobic conditions to form dykes, thus increasing oxidation of the soil C stock^22,25^. Some studies suggest that mangrove conversion results in the loss of over 50% of soil organic C and up to 90% of total ecosystem C^13,26–29^. Yet large uncertainties exist regarding the magnitude of C loss, and the implications for natural CO_2_ sinks and C reservoirs globally^30^.

Many of the shrimp ponds created in Thailand during the 1980s–2000s have proved unsustainable due to disease outbreaks^20,31^. Up to 70% are now thought to be abandoned^32^. While research documenting mangrove C stock losses due to land-use change has been steadily growing over the past half-decade^13,21,26,27,29,33,34^, little attention has been paid to understanding the fate and stability of the remaining C pools (previously sequestered and stored C) following pond abandonment (but see^24^).

When ponds are abandoned and are no longer being flooded, the C that was once buried under saturated and anoxic conditions may be released to the atmosphere (in the form of CO_2_) due to accelerated oxidation and erosion of soil organic matter^6,21^. Carbon emissions from shrimp ponds and cleared mangrove soils have been reported in earlier studies by^25^ and^22^. The oxidation and subsequent degradation of soil C following clearing and draining of peat soils has also been documented in terrestrial tropical peatlands in Southeast Asia^35^. Following wetland disturbance, it is believed that the rate of C release is most rapid during the immediate years and diminishes with time^22,36^. However, in mangroves, this process is poorly understood.

Accurate quantification of C losses or gains due to land-use change is critically important in the context of climate change, and for the inclusion of mangroves in climate change mitigation projects that require estimation of ecosystem value, such as under the United Nations Framework Convention on Climate Change program^37^.

In the present study, we quantified ecosystem C stocks of a mangrove forest (*n* = 7 transects) and abandoned shrimp ponds (*n* = 12) on an island situated within the Krabi River Estuary (Koh Klang; 7.78° N, 99.08° E), on Thailand’s southern Andaman Sea coast. Mangrove forests are an important intertidal habitat in this region^38^. In 2015, mangrove forests in Krabi province covered 32,360 ha, representing 15 percent of total mangrove cover in Thailand^39^. The overarching aim of the study was to understand the impact of shrimp farming and shrimp pond abandonment on mangrove ecosystem carbon stocks.

Ecosystem C stocks were assessed using biometric and soil coring methods along transects to determine aboveground (tree) and belowground (root + soil) C pools (see Methods section). Using a 22-year chrono-sequence approach, we also aimed to assess whether, and at what rate, C stocks were recovering after ponds had been abandoned. Abandoned ponds of different ages (10–22 years) were compared with natural reference mangrove sites. Sampled ponds had been abandoned for 10 (*n* = 3), 15 (*n* = 3), and 22 years (*n* = 3). In addition, three abandoned ponds under Ecological Mangrove Restoration (EMR)^40,41^ projects (‘EMR’; *n* = *3*) were sampled, in order to examine the impact of rehabilitation of abandoned shrimp ponds on ecosystem C stocks.

The sampled EMR ponds were designated as Community Based Ecological Mangrove Restoration (CBMER) sites. These sites had undergone hydrological modification to assist natural recruitment of mangroves into the ponds. Pond alterations during the EMR process include breaching of ponds walls, manual construction of tidal channels, and assisted dispersal of mangrove propagules (see MAP^42^, Lewis^40^ and Lewis and Brown^43^ for a full description of the EMR methodology). At the time of sampling, all EMR sites were in their third year following the completion of ecological-hydrological intervention. The ponds had been abandoned for 12–18 years before the restoration activities started.

All of the sampled abandoned shrimp ponds were formerly mangroves until they were converted in the late 1980s–1990s. The abandoned ponds had retained their basic structure at the time of sampling in April 2017, but due to removal or erosion of the old sluice gates, they were open to tidal flushing at varying levels. The pond sites were assumed not to be propagule-limited due to evidence of regrowth and their proximity to the surrounding mangroves.

## Results and Discussion

### Ecosystem carbon storage

The mean ecosystem C stock in the undisturbed mangrove forests was 1,029.5 ± 100.96 t C ha^−1^ (mean ± 1 standard error (s.e.m.)). This value is similar to ecosystem C stocks reported for estuarine mangroves in the Indo-pacific region (1,074 t C ha^−1^ ^6^), but slightly higher than the average for mangroves worldwide (965 t C ha^−1^ ^2^). The soil C stock was the largest component, representing 91.8% of the total ecosystem C.

**Figure 1:**
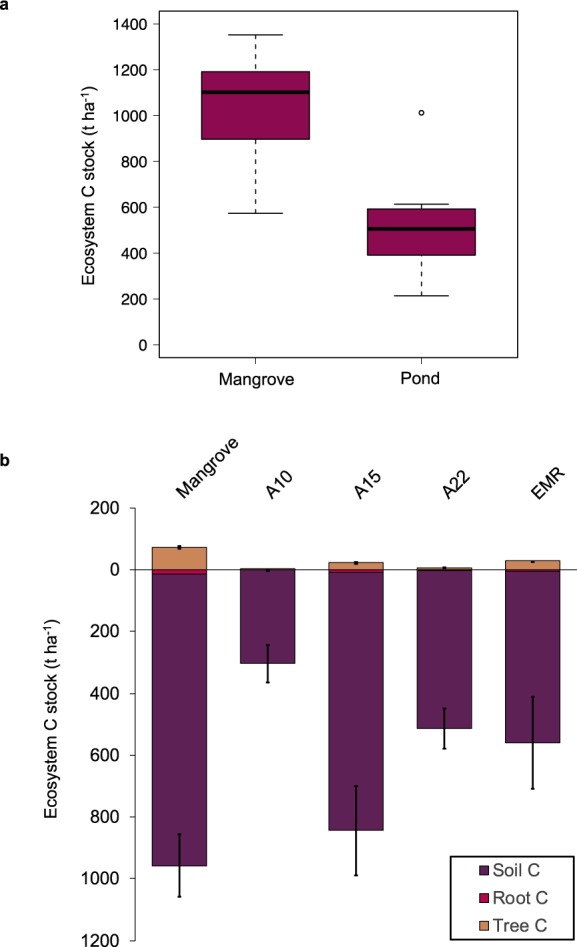
Total Ecosystem C stock of mangroves and abandoned shrimp ponds. (**a**) The mean value for all mangrove forest sites compared to the mean of all pond sites. (**b**) Ecosystem C stocks separated into aboveground and belowground C for the mangrove sites and sites of each pond category. Mangroves contained on average 1,029.5 t C ha^−1^ and ponds contained on average 541.65 t C ha^−1^. Mean total ecosystem C stocks of the pond sites was significantly lower than the mangrove sites (*p* = 0.0015).

Whole ecosystem C stocks of the abandoned pond sites (541.65 ± 79.08 t C ha^−1^) were on average 52% lower than the mangrove forests. However, the estimates were highly variable (Fig. 1). The most significant loss in ecosystem C stock was recorded for ponds sampled 10 years after abandonment (mean: 304 ± 61.3 t C ha^−1^; *p* = 0.002; 95% CI, 183–424). Ecosystem C stocks were 70% lower in these ponds compared to the undisturbed mangrove sites. This is similar to the C losses reported for mangrove conversion to shrimp ponds by^27^. By contrast, ponds sampled 15 years after abandonment had whole ecosystem C stocks (mean: 865.80 ± 146.7 t C ha^−1^) that were not significantly different to the undisturbed mangrove forest sites (*p* = 0.81). Although, because these ponds had already lost ~1.5 m of soil through pond construction, it is likely that actual carbon losses are substantially greater.

Carbon stored in the living biomass, including tree and root biomass (mean: 84.76 ± 5.16 t C ha^−1^), of the mangrove forests was much greater than tree and root C pools for the abandoned pond sites (mean: 17.22 ± 4.55 t C ha^−1^). The most recently abandoned ponds had the lowest C biomass (trace), and the ponds abandoned for 15 years had the highest (mean: 30.61 ± 3.45 t C ha^−1^), except for the EMR project ponds which were undergoing active restoration (mean: 34.31 ± 2.68 t C ha^−1^).

### Soil carbon storage

Soil depth in the undisturbed mangrove forest sites often exceeded 3 m (mean depth: 267 ± 12.53 cm). Soil depth was more variable across pond sites, ranging from 129–249 cm (mean: 180.83 ± 16.90 cm). Soil depth was a strong driver of the variations in total ecosystem C stocks across sites (*R*^2^ = 0.85, *p* < 0.001) because belowground C pools dominated at all sites, accounting for over 90% of total C stored. This finding is similar to other mangroves worldwide^6,7,44^.

Mangroves were found to be storing substantial C pools in the soils belowground, with a mean of 944.72 ± 101.5 t C ha^−1^. This estimate of soil C stocks is marginally lower than values reported for mangroves found in the Dominican Republic (1,136 t C ha^−1^ ^26^) but similar to the higher range soil C pools reported for Indonesian mangrove forests (572–1,059 t C ha^−1^ ^13^). However, because only C stocks in the uppermost 3 m of soil was estimated, the absolute ecosystem C stocks may have been underestimated^45^.

On average, the abandoned shrimp ponds contained 550.78 ± 74.62 t C ha^−1^ within the soils, which was around 40% lower than the undisturbed mangrove forests. However, soil C stocks in the most recently abandoned ponds (mean: 303.99 ± 61.24 t C ha^−1^) were over 65% lower than the mangroves. The estimates of soil C stored in abandoned ponds are higher than values in other studies (mean: 95 t C ha^−1^ ^26^; mean: 352 t C ha^−1^ ^27^). Although^27^, report a 54% loss of belowground C pools upon conversion of mangroves to shrimp ponds, which is a similar magnitude of loss recorded for the most recently abandoned ponds in the present study. By contrast, high soil C stocks were found in ponds abandoned for 15 years (835 ± 144.72 t C ha^−1^) because of their greater soil depth.

Relatively high soil C stocks in the abandoned ponds are likely driven by soil depth as some of the ponds had C-rich soils greater than 2.5 m deep, and soil C stock was found to be highly correlated to soil depth (*R*^2^ = 0.82, *p* < 0.0001). Whereas, soil depth of abandoned shrimp ponds was markedly lower in other studies (~70 cm^26,28^). Furthermore, mangrove trees had colonised in some of the ponds, and seedlings had established in all of the ponds. Across all sites, soil C stocks through the profile and in the top 15 cm soil layer was positively correlated to C stocks in the vegetation (trees + roots) (*R*^2^ = 0.43, *p* = 0.005; *R*^2^ = 0.59, *p* < 0.002, respectively).

**Figure 2:**
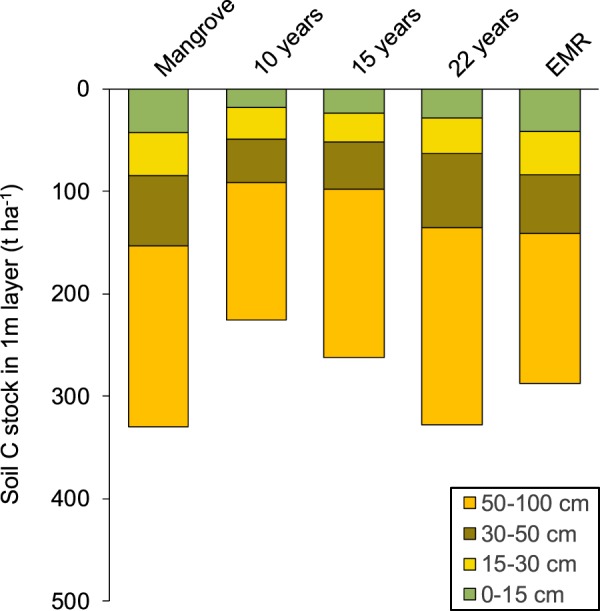
Soil C stock (t C ha^−1^) within each depth interval down to 1 m. Data shows means for the mangrove sites and the four pond categories.

Standardization of the soil profile to 1 m sediment depth allows for a better comparison of C stocks among sites because it removes the effect of soil depth on the observed variability in soil C stocks. When standardised to 1 m depth, a positive recovery trajectory for C stocks in the top 1 m soil profile is observed with age of abandoned pond (Fig. 2). Moreover, 22 years after pond abandonment, the mean soil C stock in the top 1 m of pond soil (327.6 ± 8.64 t C ha^−1^) was very similar to that of the mean soil C stock found in the top 1 m of mangrove soil (329 ± 7.55 t C ha^−1^).

**Figure 3:**
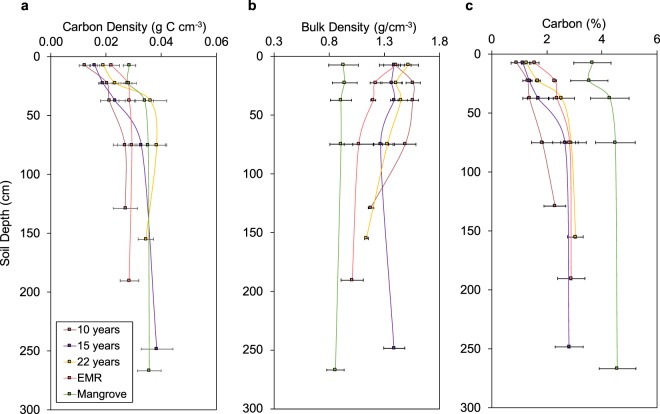
Changes in the soil properties (mean ± 1 standard error (SE)) of the mangroves and the four pond categories with depth. (**a**) Soil C density. (**b**) Soil bulk density. (**c**) Soil C concentration (%).

In terms of soil C stocks, the shallower soil depth in some of the pond sites was counteracted by higher soil bulk density (mean: 1.34 g cm^3^) compared to the mangrove sites (mean: 0.91 g cm^3^). Bulk density did not differ much through the mangrove soil depth profile, ranging from 0.86 to 0.94 g cm^3^, but was greatest in the surface soil layers of the ponds and showed a decreasing trend with depth (Fig. 3). Standing water and the use of machinery in the ponds during pond construction and use may have affected bulk density in the surface soils through compaction. Furthermore, absence of vegetation in the ponds reduces biological activity and water permeability and can lead to collapsing of the soils and greater soil compaction. Relatively high soil bulk density is also reported for abandoned ponds in the Dominican Republic (>1.27 g cm^3^ ^26^), and India (>1.0 g cm^3^ ^28^).

### Mangrove regrowth and recovery of soil carbon stocks

**Figure 4:**
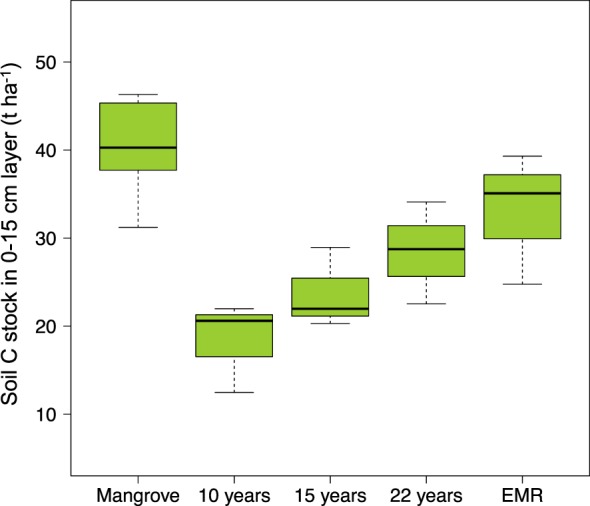
Soil C stock (t C ha^−1^) in the 15-cm soil layer. Data shows means for the mangrove sites and the four pond categories.

Along the studied chrono-sequence of abandoned pond sites, the effect of land-use change on C pools was most substantial in the near surface soil layer (0–15 cm depth), and the data also supports a positive developmental trajectory for C pools in the upper soil layer. In ponds most recently abandoned, C stored in the 0–15 cm soil layer (mean: 18.34 ± 2.97 t C ha^−1^) was 55% lower compared to the mangrove forest sites (mean: 42.49 ± 3.44 t C ha^−1^; *p* = 0.003; 95% CI, 35.7–49.2). However, in ponds abandoned for 15 and 22 years, C stocks in the same soil layer were 45% (mean: 23.74 ± 2.64 t C ha^−1^; *p* = 0.018; 95% CI, 18.6–28.9) and 33% (mean: 28.46 ± 3.34 t C ha^−1^; *p* = 0.097; 95% CI, 21.9–35.0) lower than the mangroves, respectively (Fig. 4). There was a positive linear relationship between C stocks in the 0–15 cm soil layer of ponds not under restoration and time since pond abandonment (*R*^2^ = 0.40, *p* = 0.039). As the most recent abandoned ponds were sampled 10 years after abandonment, the observed pattern suggests that C loss soon after pond abandonment (i.e. in the period 0–10 years) may have been even greater than 55%. Furthermore, when comparing carbon stocks in the top 0–15 cm of soil in the most recently abandoned ponds (mean: 18.34 ± 2.97 t C ha^−1^) with the carbon stocks at 1.5 m depth in the mangrove forest (mean = 58.82 t C ha^−1^ for depth 150–165 cm), which is likely a more just starting comparison given that ~1.5 m of soil had been removed from the ponds during construction, soil carbon stocks in the ponds is around 70% lower than the mangrove forest.

In addition, C concentration in the surface soil layer (0–15 cm) of the abandoned ponds was significantly lower in ponds most recently abandoned (mean: 0.92 ± 0.21%C; *p* = 0.033; 95% CI, 0.5–1.3) and ponds abandoned for 15 years (mean: 1.14 ± 0.05%C; *p* = 0.04; 95% CI, 1.04–1.2), compared to the mangrove forest sites (mean: 3.66 ± 0.68%C). However, C concentration in the surface layer of ponds abandoned for 22 years, and ponds under EMR projects was not significantly different to the mangrove soils (*p* = 0.07 and *p* = 0.13, respectively). Thus, the upper soil layer (0–15 cm) of ponds under restoration and those abandoned for 22 years resembled that of the natural mangrove sites.

**Figure 5:**
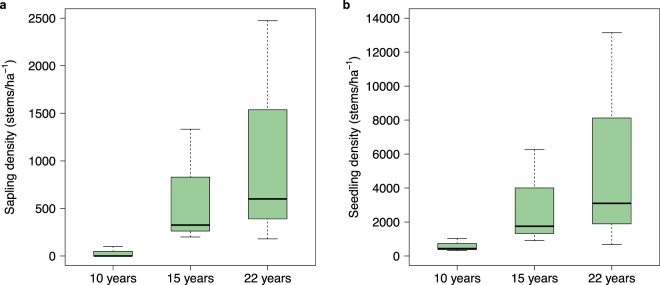
Density of (**a**) saplings, and (**b**) seedlings recorded at three of the pond categories (abandoned 10 years, 15 years, 22 years).

All of the abandoned ponds had some evidence of natural mangrove regrowth but at differing degrees. Seedling and sapling density were both highest in the ponds abandoned for 22 years (seedling density: 5,643 ± 3,817 stems/ha^−1^; sapling density: 1,085 ± 706 stems/ha^−1^) and lowest in the ponds most recently abandoned (seedling density: 597.67 ± 220 stems/ha^−1^; sapling density: 33 ± 33 stems/ha^−1^; Fig. 5). As soil C stocks are known to increase with forest age^46^, the data indicates that as mangrove trees colonise abandoned ponds, they contribute to the soil C building process. This is notable because other studies of C stock changes in abandoned ponds^26^ report no aboveground C biomass in the sampled ponds.

Our findings suggest that C accumulates in soils of the abandoned ponds over time, implying that abandoned pond areas can develop as C sinks without active restoration efforts. This could either be related to increased C burial in the pond sediments as primary production and deposition of organic matter increases with age of the regenerating mangroves^2,46^, or stabilisation of the rate of C released due to oxidation of soil organic matter with time^22^. report high short-term CO_2_ emissions from disturbed mangrove soils which decline logarithmically over a 20-year period. Stabilisation of C loss around 20 years after conversion has also been reported for marshland soils in northeastern China^47^. However, it should be emphasised that the ponds sampled in the present study had lost on average ~400 t ha^−1^ of soil C, which would likely take millennia for it to be regained.

Carbon stocks in the surface soil layer (0–15 cm) of ponds under active restoration (33.05 ± 4.32 t C ha^−1^) was not significantly different to that of the mangrove forest sites (*p* = 0.38) and ponds abandoned for 22 years (*p* = 0.93). Relatively high surface soil C pools in these ponds may be explained as C stock development due to increased tidal flooding after pond modification. Other studies support this finding. For example^48^, reported a twofold increase in the surface 5 cm soil C stocks 10 years following hydrological restoration of abandoned shrimp ponds. Furthermore, in created mangrove wetlands^49^, reported an age-related trajectory for soil C pools in the upper 10 cm, and equivalent C pools to natural mangrove sites 20 years following wetland creation. Physical disturbance of the pond soils during ecological-hydrological restoration may also stimulate nutrient release from the soil organic matter, encouraging plant growth (and hence soil C deposition). This may be particularly true where nutrients are added to ponds during pond use, and so residues (especially if additions include P) could be stimulating later tree growth. The results may then suggest that ecological-hydrological modification of abandoned ponds can provide important C storage functions as early as 3 years after restoration. It must be stressed, however, that the success of EMR relies on consideration of key biophysical processes, including propagule availability in the local area of the restoration site, elevation of the restoration site relative to the tide level (the land must be high enough above sea level so that propagules dispersed into the restoration site do not drown), and suitable hydrological conditions for propagules to settle without being dislodged by waves and currents^43^.

### Stabilisation of carbon in the sub-surface soil layers

The soil C stocks in the sub-surface soil layer (15–100 cm depth) of ponds abandoned for 10 and 15 years were not significantly different to the mangrove forests. This indicates that near surface C pools are most susceptible to land-use change, and the stability of organic C in the soils may be maintained below 15 cm depth. This finding is similar to that reported for land-use change in upland forests and marshlands^47^. For example, in marshland soils of northeast China^47^, reported a 60% loss of near surface (0–20 cm depth) C stocks within the first 15 years after conversion to cropland, but only 37% C loss in the soils between 20–40 cm depth.

Spatial differences in C stocks in the abandoned ponds may also be influenced by other confounding factors which are not controlled in natural observational studies, such as this study. For example, local differences in environmental setting and conditions, such as dominant hydrodynamic processes, landforms and vegetation, sediment supply, elevation, drainage, tidal flooding, and time when tidal inundation resumed in the ponds^2,24,50,51^.

## Conclusions

This study adds to the growing literature on the role of mangroves as highly significant global C sinks and improves understanding of C dynamics associated with land-use change in mangroves. Substantial C losses are shown to be associated with mangrove conversion for shrimp farming. However, this study demonstrates that C is preserved in deeper soil layers of some abandoned ponds, and that C accumulates in the surface soil layer after pond abandonment. This suggests that C sequestration capacity of the ecosystem may improve in abandoned shrimp ponds over time as mangroves re-establish, and that the C stored in the surface soils of ponds may be comparable to natural mangrove forests 22 years after ponds are abandoned.

## Methods

### Study design and data collection

**Figure 6:**
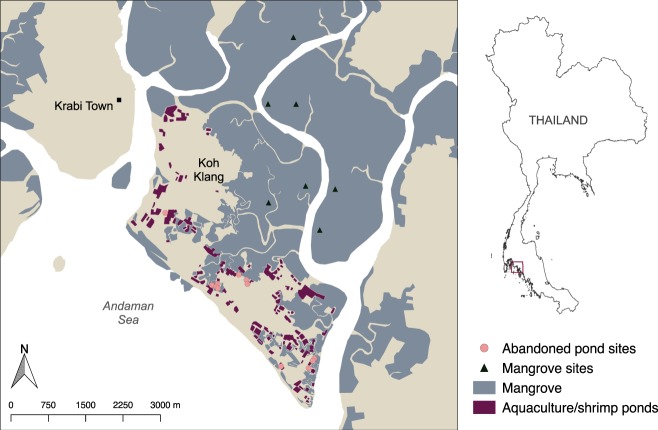
Map of the study area, Koh Klang (Krabi Province). Displayed are the location of the mangrove sites (black triangles) and abandoned shrimp pond sites (pink circles).

Forest structure, biomass, and ecosystem C stocks were determined at nineteen sites on Koh Klang, including seven secondary mangrove sites and twelve abandoned pond sites (Fig. 6; Table 1). For the mangrove forest sites, forest structure, biomass, and soil sampling was conducted in April-May 2015^52^, and additional soil sampling was conducted in May 2017. Research permission for sampling at mangrove sites was obtained from the Thai Department for Marine and Coastal Resources. Mangrove forests in the sample area have diverse species compositions, dominated by *Rhizophora*, *Xylocarpus*, and *Avicennia* species. *Avicennia* was also a dominant species present inside or around most of the abandoned ponds. Other species in or around the ponds included *Sonneratia alba*, *Rhizophora apiculata*, and *Excoecaria agallocha*. Field sampling in abandoned ponds took place in May 2017 with permissions from private owners.

**Table 1:** Qualitative description of the 12 abandoned shrimp ponds sampled.

Pond category	Estimated time since abandoned (years)	History of pond use
EMR	18	Shrimp farming then no activity from 1999 onwards. 2013 EPIC CBEMR hydrological restoration.
EMR	13	1984 mangrove converted; 1998–2001 pond used for shrimp farming; 2004 pond used for raising fish. 2013 EPIC CBEMR hydrological restoration.
EMR	12	2000–2005 Shrimp farming then no activity. 2015 GNF CBEMR hydrological restoration.
A10	10	Intensive shrimp ponds. No use after ponds were abandoned
A15	15	Intensive shrimp ponds. No use after ponds were abandoned
A22	22	Intensive shrimp ponds. No use after ponds were abandoned

CBEMR = Community-Based Ecological Mangrove Restoration; EPIC = Ecosystems Protecting Infrastructure and Communities; GNF = Global Nature Fund.

At each of the nineteen study sites, field methods for sampling forest structure, biomass, and ecosystem C stocks outlined by Kauffman and Donato^53^ were followed. For intact mangrove sites, seven transects were located randomly within the forest area, orientated perpendicular to, and between 23 and 360 m from, the coastline. Each transect consisted of five 7 m radius (154 m^2^) subplots, spaced at 25 m intervals, with the first subplot of each transect positioned at the point closest to the shoreline. A similar transect design was used to sample the abandoned pond sites to generate a comparable data set. Transects were positioned diagonally across each pond and each transect consisted of three to five 154 m^2^ subplots, depending on the size of the pond. Data was collected at each subplot for calculation of stand density, tree biomass, and total ecosystem C stocks, including aboveground C in the form of live and dead trees, downed wood, and soil C. From the C stock data collected, emissions arising from mangrove to shrimp pond conversion could be estimated using a C stock change approach.

Aboveground forest structure and species composition was recorded at each subplot. All tree stem diameters (standing and downed) greater than 5 cm at 1.3 m tree height, or 0.3 m above the highest prop root for *Rhizophora species* (dbh), were measured. The diameter of all saplings was recorded within nested 2 m radius (12.57 m^2^) subplots. All stems within the 2 m nested plot with a dbh less than 5 cm and height less than 1.37 m were classified as saplings. In abandoned ponds that had only a sparse cover of vegetation, the diameter of all mature trees and saplings was recorded throughout the 7 m radius subplot. All seedlings were counted within nested subplots.

Additionally, soil cores up to 2 m depth were taken from the centre of every subplot at each abandoned pond site, and at four of the five subplots for each of the mangrove sites due to funding constraints. A simple random sample was used to allocate four of the five subplots for soil coring along each of the seven mangrove transects. In total, this included 28 soil cores from mangrove sites and 48 soil cores from pond sites. Soil cores were obtained using an open-face peat auger with a 1 m length and 5 cm diameter. Initially, a 1 m soil core was extracted, and sub-samples of 5 cm were taken from the centre of each of the following four depth intervals (in cm): 0–15, 15–30, 30–50, 50–100. A further 5 cm sub-sample was obtained from a second core extracted from the 1–2 m soil layer where possible. At some abandoned pond sites, soils were <1 m in depth and therefore soil samples were only taken from the first 4 depth intervals. Absolute depth of soil to parent material was measured at 5 points within each subplot using a marked 3 m length probe. Depth estimates were limited to 3 m, but where depths were thought to exceed the probe length this was recorded as >3 m.

### Measuring above and belowground tree carbon

Tree diameters were converted to kg of dry weight of aboveground biomass via allometric equations developed for mangrove species found in close proximity to the study area (Table 2). Where no species-specific allometric equation was available, a general allometric equation was used to estimate biomass via species-specific wood-densities^54^. Belowground biomass estimates were calculated using a generic allometric equation for all species, developed by^54^. This equation was used for all tree species with the exception of *Rhizophora sp*., where a species-specific allometric equation was applied^55^.

**Table 2:** Allometric equations used to calculate tree and root biomass.

Species	AGB/tree (kg)	BGB/root (kg)	References
Avicennia marina; Avicenna alba	AGB = 0.308*D^2.11^	BGB = 1.28*D^1.17^	^59^
Bruguiera gymnorrhiza; Bruguiera cylindrica	WV = 0.0000754*D^2.5^ LB = 10^(−1.1679+(1.4914*(LOG(D))))^ WB = WV*WD*1000 AGB = LB + WB	BGB = 0.0188*D^2^*(D/(0.025D + 0.583))^0.909^	^60^
Nypa fructians	Log AGB = 0.85*LogD^2^L + 1.54	BGB = 0.199*WD^0.899^*D^2.22^	^54^
Rhizophora spp.	WV = 0.0000695*D^2.64^ LB = 10^(−1.8571+(2.1072*(log(D))))^ WB = WV*WD*1000 PRB = D > 5 cm PRB = WB*0.101 D > 5.0 < 10 cm PRB = WB*0.204 D > 10 > 15 cm PRB = WB*0.356 D > 15 > 20 cm PRB = WB*0.273 D > 20 cm PRB = WB*0.210 AGB = LB + WB + PRB	BGB = 0.00698*D^2.61^	^55,59,60^
Sonneratia alba	WV = 0.0003841*D^2.10^ LB = 10^(−1.1679+(1.4914*(LOG(D))))^ WB = WV*WD*1000 AGB = LB + WB	BGB = 0.199*WD^0.899^*D^2.22^	^54,60^
Other species	AGB = 0.251*WD*D^2.46^	BGB = 0.199*WD^0.899^*D^2.22^	^54^

AGB = Aboveground biomass; BGB = Belowground biomass D = dbh; L = Frond length; WV = Wood volume; PRB = Prop root biomass; LB = leaf biomass; WD = Wood density.

Estimates of above and belowground biomass were subsequently multiplied by 0.48 for aboveground and 0.39 for belowground, to convert kg of biomass to kg of C^53^. Biomass of saplings was calculated as an average value using the relationship between biomass and height of stems, based on the average sapling height. Total sapling biomass for each subplot was then calculated by multiplying the average biomass value by the number of saplings recorded in each subplot^52^.

### Measuring soil carbon

All soil samples were analysed for bulk density and percent organic matter at the Faculty of Forestry, Kasetsart University, Bangkok. Bulk density was determined as dry weight per unit volume, whereas organic matter (%OM) was determined via the percent loss on ignition technique (referred to as % LOI). Weight loss of soil samples was measured after heating subsamples for 12–24 hrs at 105 °C to remove water, and at 550 °C for four hours to remove organic matter^56^. The percentage of organic matter in the subsample was then calculated using the following equation:

$$ \% \,{\rm{LOI}}=[({\rm{dry}}\,{\rm{mass}}\,{\rm{before}}\,{\rm{combustion}}\,({\rm{g}})-{\rm{dry}}\,{\rm{mass}}\,{\rm{after}}\,{\rm{combustion}}\,(g))/{\rm{dry}}\,{\rm{mass}}\,{\rm{before}}\,{\rm{combustion}}\,(g)]\ast 100$$

Soil organic carbon (%OC) was subsequently estimated for each sample by dividing organic matter values by a factor of 2.06^53^. Soil OC density was then determined as the product of percent organic C and bulk density values. Carbon density values were combined with plot mean soil depth measurements to estimate soil C stocks for each site.

It should be noted that as soil subsamples were only taken from soil cores down to 2 m (at five depth intervals (in cm): 0–15, 15–30, 30–50, 50–100, 100–200), this meant that the deepest soil sample was taken from the 1–2 m soil layer. The C stock values for the deepest layer was then calculated as the C density from the soil sample obtained from the 100–200 cm layer multiplied by the maximum soil depth for a given site. The mean soil depth at 6 of the 7 mangrove sites and 4 of the 12 abandoned pond sites was over 2 m. As a result, where soil depth was greater than 2 m, the soil C stock was extrapolated from the 100–200 cm layer down to the maximum soil depth, thus making the assumption that there is no change in the soil C stock with depth from 2 m. Consequently, soil C stocks may have been underestimated or overestimated at sites where soil depth exceeded 2 m.

### Data analysis

All statistical analyses were computed in R Program Statistical software. Differences were considered to be significant if p ≤ 0.05. Statistical differences in mean C pools among mangrove forest and abandoned shrimp pond sites were tested using a Kruskal-Wallis rank sum test followed by a Dunn multiple comparison test to determine the differences between groups. Soil samples (n = 3–5) taken from the same pond and same soil layer were pooled and subsamples were taken for laboratory analyses. The mean C stock of a specific layer for a specific pond category was derived from averaging the mean of the layer subsamples across the ponds within the specific pond category. Therefore, when statistically comparing soil C stocks across soil layers within one pond category, the soil samples of any specific depth were not assumed to be closely linked to its upper/lower samples and were thus considered to be independent samples.

Relationships between response variables (such as C pools) and factors influencing the response (such as time since pond abandonment, soil depth, and aboveground C stocks) were tested using linear regression models (using the lm function in R). Power analysis of correlation was computed in R “pwr” package^57^ (see Supplementary Information)^58^.

## Supplementary information

Supplementary Information
