# Supplementary material for: Preservation and recovery of mangrove ecosystem carbon stocks in abandoned shrimp ponds
Source: Sci Rep. 2019 Dec 4;9:18275. doi: 10.1038/s41598-019-54893-6 (PMC6892841; doi:10.1038/s41598-019-54893-6)
Supplement: Supplementary file 1 — Supplementary Information [file 41598_2019_54893_MOESM1_ESM.pdf]

Supplementary Information

Preservation and recovery of mangrove ecosystem carbon stocks in abandoned shrimp ponds

Angie Elwin[1*], Jacob J. Bukoski[2], Vipak Jintana[3], Elizabeth J. Z. Robinson[4], Joanna Clark[1]

Affiliations:

[1] Department of Geography and Environmental Science, University of Reading, Reading, UK

[2] Department of Environmental Science, Policy and Management, University of California, Berkeley

[3] Faculty of Forestry, Kasetsart University, Bangkok, Thailand

[4] School of Agriculture, Policy and Development, University of Reading, UK

Corresponding author:

[*] angie.elwin@reading.ac.uk (Angie Elwin)

Table 1. Soil properties and aboveground and belowground carbon stocks of the mangrove and abandoned shrimp pond sites. Data are means.

		Soil Properties				C stocks (t C ha^{-1})			
**Category**	**Plot**	**Mean depth (cm)**	**C (%)**	**BD (g cm^{-3})**	**C density (g C cm^{-3})**	**Soil**	**Tree**	**Root**	**Total Ecosystem**
**Mangrove**	Mangrove J1	266	1.99	1.34	0.025	711.3	68.9	10.3	790.5
	Mangrove J2	281	4.71	0.65	0.031	1017.8	74.2	10.2	1102.1
	Mangrove J3	290	6.06	0.63	0.036	1100.9	85.7	15.4	1202.0
	Mangrove J4	199	2.39	1.08	0.025	490.9	70.4	12.5	573.8
	Mangrove J5	255	6.06	0.76	0.044	1086.3	79.5	16.0	1181.7
	Mangrove J6	283	2.57	1.13	0.028	912.3	77.7	13.6	1003.6
	Mangrove J7	296	4.97	0.79	0.038	1293.6	48.0	11.0	1352.6
**Mangrove mean**		267	4.11	0.91	0.032	944.7	72.06	12.70	1029.5
**A10**	A10 P4	121	1.20	1.41	0.017	214.2	T	T	214.24
	A10 P5	149	1.84	1.39	0.025	421.1	T	T	421.10
	A10 P6	117	1.60	1.51	0.023	276.7	T	T	276.68
**A10 mean**		129	1.55	1.43	0.022	304.0	T	T	304.01
**A15**	A15 P2	240	1.26	1.45	0.018	545.8	22.7	3.92	572.4
	A15 P3	265	2.28	1.25	0.028	974.8	25.5	11.97	1012.3
	A15 P3b	241	2.26	1.38	0.031	984.9	19.2	8.58	1012.7
**A15 mean**		249	1.93	1.36	0.026	835.2	22.5	8.16	865.80
**A22**	A22 P7	158	2.36	1.36	0.031	537.8	5.89	2.66	546.34
	A22 P8	182	2.33	1.37	0.031	605.2	5.45	2.90	613.51
	A22 P9	126	2.14	1.37	0.028	388.0	7.59	4.46	400.04
**A22 mean**		155	2.28	1.37	0.030	510.3	6.31	3.34	519.96
**EMR**	EMR P1	275	2.22	1.30	0.029	840.6	NS	NS	NS
	EMR P10	136	2.22	1.11	0.024	346.2	27.7	9.33	383.16
	EMR P11	160	2.70	1.14	0.029	474.1	28.7	2.94	505.71
**EMR mean**		190	2.38	1.18	0.027	553.6	28.2	6.13	444.44
**Pond mean**		180.8	2.03	1.34	0.026	550.9	12.97	4.25	541.65

NS denotes where trees were not sampled; T=trace.

Bulk density (g cm^{-3})

**Category and Plot**	**Depth (cm)**					
	**0-15**	**15-30**	**30-50**	**50-100**	**100-200**	**Overall Mean (± 1SE)**
**Mangrove J1**	1.53	1.32	1.39	1.26	1.21	1.34 ± 0.06
**Mangrove J2**	0.57	0.57	0.7	0.68	0.75	0.65 ± 0.04
**Mangrove J3**	0.55	0.68	0.66	0.62	0.65	0.63 ± 0.02
**Mangrove J4**	1.09	1.27	0.97	1.1	0.95	1.08 ± 0.06
**Mangrove J5**	0.78	0.7	0.8	0.77	0.76	0.76 ± 0.02
**Mangrove J6**	1.19	1.07	1.06	1.23	1.08	1.13 ± 0.04
**Mangrove J7**	0.82	0.89	0.81	0.7	0.74	0.79 ± 0.03
**Mangrove mean**	0.93	0.94	0.91	0.91	0.86	0.91 ± 0.01
**A10 P4**	1.19	1.53	1.65	1.44	1.22	1.41 ± 0.09

**A10 P5**	1.33	1.68	1.47	1.35	1.14	1.39 ± 0.09
**A10 P6**	1.67	1.46	1.55	1.68	1.19	1.51 ± 0.09
**A10 mean**	1.40	1.56	1.56	1.49	1.18	1.44 ± 0.07
**A15 P2**	1.56	1.33	1.44	1.41	1.53	1.45 ± 0.04
**A15 P3**	1.24	1.24	1.4	1.18	1.21	1.25 ± 0.04
**A15 P3b**	1.36	1.55	1.33	1.23	1.44	1.38 ± 0.05
**A15 mean**	1.39	1.37	1.39	1.27	1.39	1.36 ± 0.02
**A22 P7**	1.62	1.33	1.44	1.31	1.12	1.36 ± 0.08
**A22 P8**	1.59	1.39	1.29	1.42	1.17	1.37 ± 0.07
**A22 P9**	1.34	1.5	1.61	1.26	1.12	1.37 ± 0.1
**A22 mean**	1.52	1.41	1.45	1.33	1.14	1.37 ± 0.07
**EMR P1**	1.42	1.28	1.25	1.34	1.19	1.30 ± 0.04
**EMR P10**	1.34	1.12	1.17	0.94	1.0	1.11 ± 0.07
**EMR P11**	1.47	1.26	1.19	0.93	0.84	1.14 ± 0.11
**EMR mean**	1.41	1.22	1.20	1.07	1.01	1.18 ± 0.07
**Pond mean**	1.42 ± 0.04	1.39 ± 0.05	1.4 ± 0.05	1.29 ± 0.06	1.18 ± 0.05	1.34 ± 0.05

C content (%)

**Category and Plot**	**Depth (cm)**					
	**0-15**	**15-30**	**30-50**	**50-100**	**100-200**	**Overall Mean**
**Mangrove J2**	4.98	4.04	4.42	4.77	5.35	4.71
**Mangrove J3**	5.77	5.60	6.52	6.22	6.20	6.06
**Mangrove J4**	1.95	1.90	2.96	2.41	2.71	2.39
**Mangrove J5**	5.53	5.96	6.57	6.58	5.66	6.06
**Mangrove J6**	2.14	2.10	2.77	2.72	3.13	2.57
**Mangrove J7**	3.68	3.66	4.87	6.16	6.49	4.97
**Mangrove mean**	3.66	3.54	4.29	4.49	4.56	4.11
**A10 P4**	1.15	0.98	0.88	1.43	1.54	1.20
**A10 P5**	1.10	1.41	1.59	2.60	2.52	1.84
**A10 P6**	0.50	1.53	1.65	1.48	2.82	1.60
**A10 mean**	0.92	1.30	1.38	1.84	2.29	1.55
**A15 P2**	1.24	1.28	0.88	1.11	1.82	1.26
**A15 P3**	1.09	1.41	2.09	3.44	3.34	2.28
**A15 P3b**	1.08	1.42	2.08	3.43	3.28	2.26
**A15 mean**	1.14	1.37	1.68	2.66	2.81	1.93
**A22 P7**	0.93	1.60	3.25	2.64	3.36	2.36
**A22 P8**	1.43	1.85	2.70	3.20	2.47	2.33
**A22 P9**	1.43	1.55	1.65	2.79	3.29	2.14
**A22 mean**	1.26	1.67	2.53	2.88	3.04	2.28
**EMR P1**	1.65	2.18	2.38	2.25	2.65	2.22
**EMR P10**	1.23	2.28	2.14	3.30	2.16	2.22
**EMR P11**	1.78	2.40	2.59	2.92	3.83	2.70
**EMR mean**	1.55	2.29	2.37	2.82	2.88	2.38
**Pond mean**	1.22	1.66	1.99	2.55	2.76	2.03

Table 2. Power analysis of correlation

**Variables**		**R^2**	**p-value**	**Power**
Soil depth	Total ecosystem C stocks	0.85	<0.001	0.84
Soil depth	Soil C stock	0.82	<0.0001	0.72
Soil C stock through soil profile	Tree + root C stock	0.43	0.005	0.16
Soil C stock in 0-15cm soil layer	Tree + root C stock	0.59	<0.002	0.34
